# Medical cannabinoids for painful symptoms in patients with severe dementia: a randomized, double-blind cross-over placebo-controlled trial protocol

**DOI:** 10.3389/fpain.2023.1108832

**Published:** 2023-05-24

**Authors:** Federica Bianchi, Sophie Pautex, James Wampfler, François Curtin, Youssef Daali, Jules Alexandre Desmeules, Barbara Broers

**Affiliations:** ^1^ Fondation pour l’accueil et l’hébergement de personnes âgées, Long-term Care Home “les Tilleuls”, Geneva, Switzerland; ^2^ Palliative Medicine Division, Department of Rehabilitation and Geriatrics, Geneva University Hospitals, Geneva, Switzerland; ^3^ Faculty of Medicine, University of Geneva, Geneva, Switzerland; ^4^ Clinical Pharmacology and Toxicology Division, Department of Anesthesiology, Pharmacology, Intensive Care and Emergency Medicine, Geneva University Hospitals, Geneva, Switzerland; ^5^ Institute of Pharmaceutical Sciences of Western Switzerland (ISPSO), University of Geneva, Geneva, Switzerland; ^6^ Primary Care Division, Geneva University Hospitals, Geneva, Switzerland

**Keywords:** pain, dementia, behavioural troubles, long-term care facilities, cannabinoids, THC/CBD

## Abstract

**Background:**

In an observational study in Geneva (Switzerland), we found that administering a standardized THC/CBD oil was feasible, safe, and beneficial in an elderly polymedicated population with severe dementia, behavioral troubles, and pain. Those findings need to be confirmed in a randomized clinical trial.

**Objectives:**

The MedCanDem trial is a randomized, double-blind cross-over placebo-controlled trial to study the efficacy of cannabinoids in improving painful symptoms during severe dementia disorders in patients living in long-term care facilities in Geneva. This manuscript describes the MedCanDem trial protocol.

**Materials and methods:**

Participants will be patients suffering from severe dementia associated with pain and behavioral troubles and living in long-term care facilities. We selected five facilities specialized in caring for severely demented patients in Geneva (Switzerland). A total of 24 subjects will be randomized 1:1 to the sequence study intervention/placebo or the sequence placebo/study intervention. Patients will receive study intervention treatment or placebo for eight weeks, and then after a one-week wash-out, treatments will be inversed for another eight weeks. The intervention will be a standardized THC/CBD 1:2 oil extract, and the placebo will be a hemp seed oil. The primary outcome is the reduction from the baseline of the Cohen-Mansfield score; secondary outcomes include the reduction in the Doloplus scale, the reduction of rigidity, the monitoring of concomitant drugs prescription and de-prescription, the safety assessment, and a pharmacokinetic evaluation. The primary and secondary outcomes will be assessed at the baseline, after 28 days, and at the end of both study periods. In addition, safety laboratory analysis, pharmacokinetic evaluation, and therapeutic drug monitoring for the cannabinoids will be evaluated through a blood sample analysis conducted at the beginning and the end of both study periods.

**Discussion and conclusion:**

This study will allow us to confirm the clinical results observed during the observational study. It represents one of the few studies aiming to prove natural medical cannabis efficacy in a population of non-communicating patients with severe dementia, experimenting with behavioral troubles, pain, and rigidity.

**Trial registration:**

The trial has Swissethics authorization (BASEC 2022-00999), and it is registered on clinicaltrials.gov (NCT05432206) and the SNCTP (000005168).

## 1. Introduction

According to the World Health Organization, dementia affected 55 million people globally in 2020, and they are expected to climb to 78 million by 2030 (1). In Switzerland, the number of people with degenerative dementia is around 155.000, of whom 7,400 are under age 65, and about 40% live in long-term care facilities (2, 3). In Switzerland, the direct and indirect cost of dementia reached more than 11 billion in 2019, of which a large proportion (53%) was spent on institutional care (4). Behavioral and psychological symptoms of dementia (BPSD) affect up to 80% of patients with advanced dementia (5), and 97% of patients with dementia will develop at least one behavioral symptom at a certain point in their illness (6). They alter the quality of life of patients and caregivers and increase the economic burden of the disease. In addition, they lead to medication overuse and can lead to other complications of dementia: chronic pain, physical trauma, and falls (7).

Pain can be associated with the deterioration of the patient's overall condition and the onset or worsening of behavioral symptoms (8, 9). Studies suggest that more than 50% of people with dementia have underestimated and undertreated pain (10). The strong association of pain with psychological symptoms and aggressive behavior (11) suggests that optimizing pain treatment might contribute to the relief of psychological symptoms and improve patients' functions (12). A correct assessment of pain in demented patients is a challenge but validated observational staff-administered assessment scales exist, such as the Doloplus questionnaire (13, 14).

Psychological symptoms treatment consists of correcting somatic and psychiatric factors, improving environmental factors, and implementing non-medicinal psychological, psychosocial, and behavioral interventions (16). Unfortunately, despite various interventions, some patients continue to present behavioral problems, such as aggressiveness and shouting. Often, drug treatments must be introduced (antidepressant, neuroleptics, atypical antipsychotics, or benzodiazepines), with the limits of their effectiveness and side effects (17, 18) like akathisia, wrong eating habits, atypical strokes, paradoxical agitation, or accentuation of memory disorders. Unfortunately, current data in the literature do not allow us to recommend specific treatments for Alzheimer's disease to prevent and treat psychotic symptoms and disruptive behavior.

The medical use of cannabinoids is the subject of growing interest from the international scientific community. Recent publications report on the pharmacological properties of cannabinoids and their therapeutic potential in several pathologies, such as pain management, glaucoma, epilepsy, autoimmune diseases, and disorders often associated with aging (18, 19). Cannabis sativa's main cannabinoids constituents are delta-9-tetrahydrocannabinol (THC) and cannabidiol (CBD), but other cannabinoids and terpenes also are pharmacologically active (20). Most clinical studies suggest that a combination of THC and CBD have antispasmodic effects (21, 22), whereas given alone THC seems to have appetite increasing and “derealization” effects (23, 24), and CBD has more calming anti-convulsive effects (25). THC's activity mimics the effect of the endocannabinoids, especially on the CB1 receptors mainly expressed in the central nervous system. Instead, CBD activities are yet to be fully understood, appearing to interact - as an agonist or antagonist - with various receptors including CB1s and CB2s, the vanilloid type 1 channel (TRPV1) (26), the serotonin receptor 5HT_1A_ (27)_,_ and the enzymes responsible for anandamide uptake and hydrolysis (28, 29). An intriguing property of CBD might be its capacity to antagonize the psychotropic effects of THC, possibly modulating the THC-CB1 receptor interaction (30, 31). The clinical observation and the literature report that CBD co-administration can counterbalance THC psychotropic effects (32–34) and that oral THC/CBD combination results in higher tolerability and patient satisfaction (32, 35). Still, effects can be dose and context dependent, and the suggested antipsychotic effect of inhaled CBD on THC recently has been contested (36). A critical aspect adding complexity to the study of cannabis sativa and cannabinoid's medicinal properties is the possibility of the “entourage effect,” first proposed in 1998 by Mechoulam and Ben-Shabat (37). The entourage effect refers to the natural extract potentiation or balancing of activities compared to pure synthetic drugs, probably due to other cannabinoids and non-cannabinoid derivatives naturally present in the extract. Some clinical experiences suggest that the entourage effect might be involved in better clinical outcomes and fewer side effects (38, 39). This aspect and the importance of the GMP quality of the administered product, require a careful product selection to guarantee the certification, reproducibility and control of the cannabis strain and the study drug manufacturing process.

The efficacy of cannabis derivatives for chronic pain is supported by clinical trials (40, 41). Still, there is a lack of consensus (42), and a poor quality in data available in literature on the use of cannabinoids-based medicines for pain, with a great variability in terms of pain reduction results, type of cannabis derivative or synthetic drug used, dose range or length of administration. Still, in clinical practice, chronic pain is the primary indication for the prescription and usage of cannabinoids in Switzerland (43). Concerning treating dementia-related disorders, various studies support using THC and CBD. In-vitro and in-vivo studies elucidate the endocannabinoid system involvement in preventing and treating Alzheimer's disease and dementia (44). However, the efficacy of cannabinoids in reducing neuropsychiatric symptoms is still to be confirmed, and clinical trials are mainly based on cannabinoids' synthetic analogs, dronabinol or nabilone (45). Preliminary results of the few clinical trials demonstrated that cannabinoids could be safely administered to patients with dementia (32). Literature also provides moderate support for the efficacy of cannabinoids on behavioral and psychological symptoms of dementia (BPSD). Still, the lack of robust randomized clinical trials doesn't allow generalization of the findings (45, 46).

The pharmacokinetics of THC and CBD involve their metabolization by the liver p450 enzymes. The concomitant administration of medical cannabis with other drugs might result in a higher risk of drug-drug interactions, thus reducing medication activity or increasing toxicity. THC and CBD are metabolized by liver CYP450 enzymes (THC by CYP2C9, CYP2C19, and CYP3A4 isoenzymes; CBD mainly by CYP2C19, CYP1A2, and CYP3A4) (47–49). In vitro drug-drug studies confirm that there is potential for THC and CBD to have pharmacokinetic interactions, possibly affecting the metabolism or the activity of drugs that are substrates for CYP2C9, CYP2C19 and CYP1A2 (e.g., fluoxetine, clopidogrel or tizanidine) (50–52). However, the in-vitro and in-vivo observations haven't always been confirmed in humans (53). Data is still to be validated, but the few clinical studies confirm cannabinoids' implication in cytochromes induction or inhibition (54). Moreover, the route of administration, the age of patients, and the number of concomitant medications represent critical variables that might need specific clinical studies to confirm the cannabinoids' pharmacokinetics before generalizing results from other trials.

In a recent feasibility prospective observational study (55), we demonstrated that 19 patients with severe dementia and significant behavior problems could receive a natural THC/CBD oral oil medication for over a year without discontinuation due to side effects. The Cohen-Mansfield Agitation Inventory score, the Neuropsychiatric Inventory score, and the rigidity score markedly improved after the cannabis administration, then stabilized and lasted throughout the observation period. In addition, half of the patients decreased or stopped other pain and psychotropic medications. The families and the caretakers appreciated the decrease in rigidity, consequently making daily care more manageable, the improved direct contact with the patients, and the improved behavior. A preliminary pharmacokinetic analysis to evaluate the possibility of drug-drug interactions detected a slight reduction in some hepatic enzymatic (CYP1A2 and CYP2C19) activity in the cohort of observed patients. Still, no severe adverse events related to the treatment or concomitant drugs were reported, and the safety profile was favorable, with no drug discontinuation due to side effects (55).

The encouraging results of the observational study, both on safety and feasibility, and the need to add knowledge on the possible use of natural cannabis oil to reduce BPSD and pain in patients with severe dementia, prompted us to set up a randomized clinical trial. This manuscript presents the trial protocol planned for 2023 in five nursing homes in Geneva, Switzerland.

This trial aims to prove the efficacy of a standardized THC/CBD natural cannabis oil in reducing the behavioral and psychological symptoms of dementia, pain, and rigidity in patients. This trial includes evaluating plasma levels of the cannabinoids and derivatives and their potential influence on the cytochromes P450 - CYP1A2, CYP2B6, CYP2C9, CYP2C19, CYP2D6, CYP3A4/5 - to bring further knowledge to the possible impact of the cannabinoids on the metabolic enzymatic activity in this patient population.

## 2. Methods and materials

### 2.1. Ethics and authorities' approval

The investigators drew up the study protocol following the SPIRIT 2013 statements and checklist. They assure they will conduct the study following the principles enunciated in the current version of the Declaration of Helsinki, the guidelines of Good Clinical Practice (GCP) issued by ICH, and Swiss Law and Swiss regulatory authority's requirements.

The study protocol and procedures have been reviewed and approved by the Geneva Ethics Commission (number 2022-00999) and by Swissmedic, the Swiss Agency for therapeutic products. This trial has been registered on Clinicaltrials.gov: NCT05432206. The purpose, nature, and potential risks of this trial will be fully explained to the participants’ relatives or to representatives in the medical field (art 378 Swiss Civil Code) who will provide written informed consent before participating.

The results will be the object of scientific publications and communication to scientific conferences. Any reference to the identity of the study participants will be avoided, and confidentiality will be guaranteed.

### 2.2. General study design

**Figure 1:**
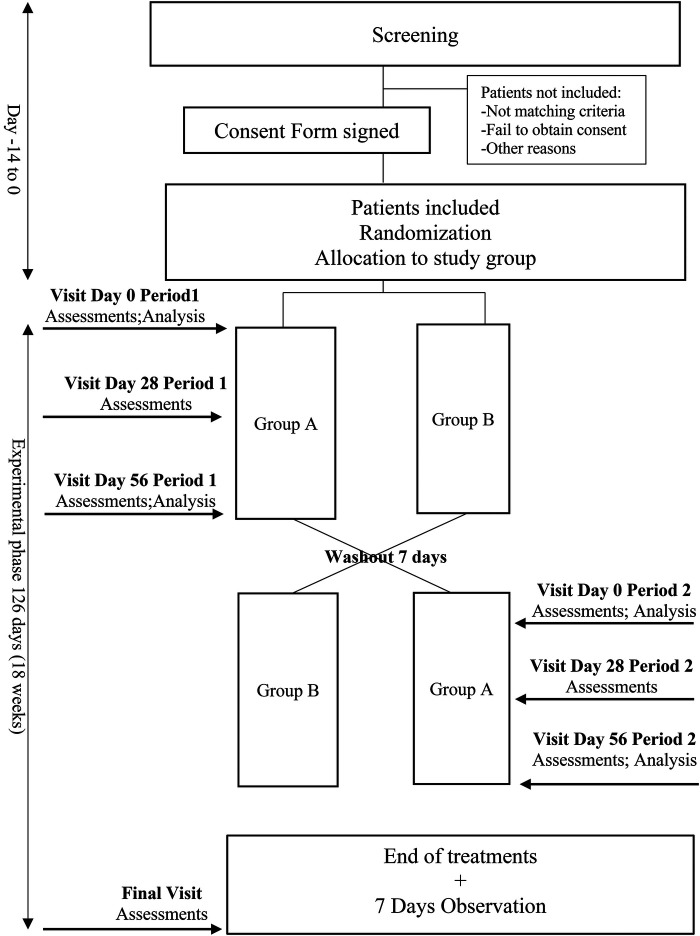
Study flow chart of the MedCanDem trial, medical cannabinoids for painful symptoms in patients with severe dementia living in home-care facilities.

This study is designed as a randomized, double-blind, placebo-controlled AB/BA cross-over trial. Two weeks are planned for the inclusion of patients. After inclusion and randomization, the investigational medicinal drug or the placebo will be administered for eight weeks. A 7-day wash-out period will follow, with no drug administration. Afterward, the treatments will be inversed and administered for eight weeks so that all patients can access the study drug. An observation week, without treatment intake, is planned at the end of the second period (Figure 1).

### 2.3. Setting

Patients will be included in five long-term home facilities in Geneva (Résidence Butini, Résidence de la Rive, Les Charmettes, Résidence Fort-Barreau, Résidence Les Tilleuls), all specialized in the care of severely demented patients. There will be no stratification between the study settings.

Two permanent staff members for each facility will be the local referents for the study and will be trained by the study staff for all procedures. They will supervise the daily treatment administration and participants' daily surveillance and maintain constant contact with the study staff. Appropriate training will be provided to all personnel involved in the direct care of patients, and all the facilities staff will be informed of the study and the contact persons in case of need.

### 2.4. Population

The population will be male and female subjects, 55 years of age and over, suffering from severe dementia and behavioral problems, and permanent residents in a long-term care facility. The gender distribution will reflect the facilities' population, and a majority of females might be expected. Patients' mental incapacity will be assessed before inclusion, and informed consent must be obtained from a representative in the medical field (art 378 Swiss Civil Code). If patients show signs and symptoms of unwillingness to participate, they will be excluded from participation.

Inclusion criteria:

• Age ≥55
• Clinical Dementia Rating [35] ≥3
• Neuropsychiatric Inventory [NPI] score >10, notwithstanding optimal conventional treatment.
• Fully vaccinated for Covid-19 and/or recovered from Covid-19 for at least two weeks at the time of inclusion. (The study started to be planned in May 2021, when the SARS-CoV-2 outbreak was a major concern and vaccine of recent introduction. We added this inclusion criteria to avoid complications to the study procedures – no interference or complication is expected if patients get sick during the trial)

Non-inclusion criteria:

• Severe organ deficiencies such as cardiac, pulmonary, hepatic, renal insufficiency
• Unstable heart rhythm
• Symptomatic orthostatic hypotension
• Patients presenting significant changes or instability of psychotropic medication
• Patients taking a THC or CBD treatment in the week preceding the study
• Patients presenting any other medical conditions that would prevent the participation in the whole study protocol

### 2.5. The investigational products

The investigational medicinal drug will be a standardized cannabis sativa full extract from defined cannabis varieties formulated in hemp seed oil. The oil contains 11 mg THC/g and 22 mg CBD/g, THC/CBD (ratio 1:2), odor masked. The placebo will be a hemp seed oil, <0.1 mg THC/g and <0.1 mg CBD/g, odor masked, color adjusted. The cannabis sativa oil extract and the placebo are provided by Cannapharm AG. Cannapharm AG holds all the GMP certifications for the product and will be responsible for the product characteristics throughout the study.

The dose rationale is based on our clinical experience over the last years and the prescription schema of nabiximols, the only comparable cannabis medicine authorized in Switzerland. The initial dose of 2,5 mg THC/ 5 mg CBD will be individually titrated up to a maximum of 20 mg THC/ 40 mg CBD divided into two administrations per os daily (Table 1). The proposed dosage is under the dosage suggested in the package insert for Sativex® (around 30 mg THC per day after the adaptation phase). The study investigators will decide the dose adjustments on daily individual evaluation. If patients refer to unpleasant symptoms or an adverse event is observed, the dosage will be evaluated, and the titration will be put on hold until stabilization. If an effect is remarked, but subsequent dosage augmentation does not produce improvements, the titration will be stopped, and the dosage will be fixed at the last time the change was observed. Particular attention will focus on blood pressure that should remain within a 15% range compared to patients' average blood pressure, based on the last seven days' medical history. In case of systolic and/or diastolic blood pressure variations, the titration will be stopped, and the dosage will be evaluated. Titration will be continued after blood pressure stabilization. The health professionals in charge of the patients will continue patients' regular treatments.

**Table 1:** Study drug titration.

Day	Number of drops in the morning	Number of drops in the evening	Total drops in the day (mg THC/mg CBD)
1	0	7	7 (2.5 mg THC/5 mg CBD)
2	0	7	7 (2.5 mg THC/5 mg CBD)
3	7	7	14 (5 mg THC/10 mg CBD)
4	7	7	14 (5 mg THC/10 mg CBD)
5	7	14	21 (7.5 mg THC/15 mg CBD)
6	7	14	21 (7.5 mg THC/15 mg CBD)
7	14	14	28 (10 mg THC/20 mg CBD)
8	14	14	28 (10 mg THC/20 mg CBD)
9	14	21	35 (12.5 mg THC/25 mg CBD)
10	14	21	35 (12.5 mg THC/25 mg CBD)
11	21	21	42 (15 mg THC/30 mg CBD)
12	21	21	42 (15 mg THC/30 mg CBD)
13	21	28	49 (17.5 mg THC/35 mg CBD)
14	21	28	49 (17.5 mg THC/35 mg CBD)
15	28	28	56 (20 mg THC/40 mg CBD)

#### 2.5.1. Post-trial treatment access

The post-trial treatment access was a significant concern in setting up the clinical trial. Medical cannabis oil is available in Switzerland on physician prescription. Still, the treatment is expensive and not reimbursed by health insurance. We wanted to guarantee that physicians could continue prescribing medical cannabis oil to their patients if willing to do, without additional costs or administrative burden. Therefore, Cannapharm AG granted us medical cannabis free supply for five years after the study termination to all participants to whom the physicians would consider the prescription. In addition, the study staff will provide all safety and usage information to the physicians needing assistance.

### 2.6. Outcomes and data collection

#### 2.6.1. Primary outcome

The primary endpoint is the variation in the Cohen-Mansfield Agitation Inventory score (56). This questionnaire comprises 29 items, each evaluating the frequency of agitated behaviors with values from 1 (never) to 7 (several times an hour). The final score ranges from 29 to 203, the higher value meaning a worse situation. The CMAI is validated and broadly used to evaluate agitation in long-term facilities.

The CMAI assessments are planned on the first day, before the drug intake, after four weeks, and at the end of both study periods (Table 2). A final evaluation will occur after the observation week planned at the end of the two study periods. A trained study staff member will conduct evaluations by interviewing a panel of healthcare professionals who usually care for patients. The questionnaire will be based on the patients' behaviors the week before the assessment.

**Table 2:** Study schedule.

Period 1							
	**Screening**	**Allocation**	**Intervention**				
Days	−14/−2	−2	0	0–56	28	54	56
Patient Information and Informed Consent	X	x					
Demographics	x	x					
Medical History	x						
Drug History	x						
In- /Exclusion Criteria	x	x					
Allocation to study kit			x				
**Vital signs:**							
Pulse	x	x	x		x		x
Blood pressure	x	x	x	x	x	x	x
Temperature	x		x		x		x
Weight	x		x		x		x
Physical Examination	x						x
**Safety Lab:**							
Blood formula	x		x				x
Hemoglobin	x						
Na	x						
K	x						
Urea	x						
Creatinine	x						
Protein	x						
ASAT/ALAT	x						
Cohen Mansfield scale			x		x		x
Doloplus score			x		x		x
Rigidity scale			x		x		x
Neuropsychiatric scale			x		x		x
Most invalidating ADL			x		x		x
Most invalidating behavioural trouble			x		x		x
TGIC					x		x
FGIC					x		x
Treatment dosage adaptation Log					x		x
**Cannabinoids plasma concentration (TDM)**							
THC			x				x
11-OH-THC			x				x
THC-COOH			x				x
CBD			x				x
**Endocannabinoids blood dosage**							
Anandamide			x				x
2-arachidonyl glycerol			x				x
Preparing patients for Geneva-cocktail		x				x	
P450 activity–Geneva-cocktail			x				x
Co-medication with dosage	x		x		x		x
Co-medication dosage changings					x		x
Adverse Events					x		x
Period 2							
	Wash out	Intervention	Final Week				
Days	−2	0	0–56	28	54	56	+7
Physical Examination						x	x
**Vital signs:**							
Pulse		x		x		x	x
Blood pressure	x	x	x	x	x	x	x
Temperature		x		x		x	x
Weight		x		x		x	x
**Safety Lab:**							
Blood formula		x				x	
Cohen Mansfield scale		x		x		x	x
Neuropsychiatric scale		x		x		x	x
Rigidity scale		x		x		x	x
Doloplus score		x		x		x	x
Most invalidating ADL		x		x		x	x
Most invalidating behaviour troubles		x		x		x	x
TGIC		x		x		x	x
FGIC		x		x		x	x
Treatment dosage adaptation Log				x		x	
**Cannabinoids plasma concentration (TDM)**							
THC		x				x	
11-OH-THC		x				x	
THC-COOH		x				x	
CBD		x				x	
Endocannabinoids blood dosage							
Anandamide		x				x	
2-arachidonyl glycerol		x				x	
Preparing patients for Geneva-cocktail	x				x		
P450 activity–Geneva-cocktail		x				x	
Co-medication with dosage		x		x		x	x
Co-medication dosage changings				x		x	x
Adverse Events				x		x	x

ADL, activity daily living; TGIC, team general impression of change; FGIC, family general impression of change; THC, delta-9-tetrahydrocannabinol; CBD, cannabidiol; 11-OH-THC, 11-hydroxy-tetrahydrocannabinol; THC-COOH, carboxy-tetrahydrocannabinol; TDM, therapeutic drug monitoring.

#### 2.6.2. Secondary outcomes

##### 2.6.2.1. Clinical outcomes

The secondary outcomes will be the score changings of the Doloplus pain scale (13), the NeuroPsychiatric Inventory scale (NPI) (57), the Unified Parkinson's Disease Rating item 22 rigidity Scale (UPDRS) (58), and the changing in the most incapacitating daily activity and behavioral trouble (MIDA and MIBT). Those assessments will be held at the same time as the primary endpoint, during the interview with the healthcare professionals usually taking care of patients (Table 2).

The Doloplus pain assessment is an observational staff-administered evaluation of pain for patients with communication disorders. It is composed of 10 items, each exclusively and progressively rated from 0 to 3, for an overall score from 0 to 30, the higher score meaning the worse outcome. The NPI is a questionnaire measuring 12 neuropsychiatric symptoms in the period before assessment. It rates the frequency on a 4-grade score multiplied by the severity on a 3-grade score. Each symptom can be rated from 0 (absent) to 12 (high frequency and severe) for a final score ranging from 0 to 144, the higher score meaning the worse outcome. The UPDRS (item 22) evaluates the patients' rigidity during the passive movements of the main joints in a relaxed and sitting position. The score ranges from 0 to 4 (0 = no rigidity; 4 = extreme difficulty in movements). The score will be attributed to patients by a trained facility health care provider to avoid perturbing patients with the presence of the study staff, to which they might not be accustomed. The evaluation will occur on pre-determined days and be communicated to the study team during the interviews.

The healthcare professionals will define each participant's most incapacitating daily activity. During our observational study, we remarked that the most common difficulties were personal daily care, dressing, and undressing. Therefore, they will be assessed on a 0–10 scale, with 0 meaning no difficulty and 10 extreme complexities. Likewise, the most incapacitating daily behavior (e.g., screaming, opposition) defined by the facility staff will also range from 0 to 10, 10 meaning maximum trouble.

Additional secondary outcomes will comprise the evaluation of the impression of change after the drug intake (Table 2). At two time points, after four weeks and after eight weeks of both study periods, we will ask the families and the facility staff to express their impression of change, rating it on a visual scale ranging between −10 for the maximum worsening and +10 for the maximum bettering. Concerning the facility staff, the assessment will take place during the interviews. For the families, we will contact them for a short personal or virtual consultation that will also represent an exchange moment for the families with the study staff to ask questions or solve doubts.

Finally, we included the registration of changes in concomitant medications, with a particular interest in pain and psychotropic drugs as a secondary endpoint.

##### 2.6.2.2. Pharmacokinetic outcomes

A pharmacokinetic evaluation is planned for the first and the last day of both study periods (Table 2). Patients will receive the Geneva cocktail (59) on an empty stomach in the morning before drug intake. After two hours, a blood sample will be collected from each patient by the nursing home staff and sent to the Geneva University Hospitals' laboratory for analysis. They will measure the therapeutic dosage through the plasma concentrations of THC, its two metabolites, 11-hydroxy-tetrahydrocannabinol (11-OH-THC) and Carboxy-tetrahydrocannabinol (THC-COOH), and CBD. They will also register the plasma concentrations of endocannabinoids anandamide (AEA) and 2-arachidonylglycerol (2-AG). Also, the changings in enzymatic activity of six cytochromes p450 (1A2, 2B6, 2C9, 2C19, 2D6, 3A4/5) will be evaluated by measuring the changes in the metabolic ratio (metabolite concentration/substrate concentration) of the corresponding substrates.

##### 2.6.2.3. Safety outcomes

Our first concern is the safety of our patients, and their well-being will be constantly monitored and evaluated by the facilities staff that will refer any observation to the study team. During the entire duration of the study, all adverse events (AE) and all serious adverse events (SAEs) are collected, thoroughly investigated, and documented in source documents. Due to the status of our population, we expect adverse events unrelated to the investigational product. Therefore, AE related to the investigational drug, SAE, and suspected unexpected serious adverse reactions (SUSAR) would be reported and documented in the study safety outcomes. The collected information will include event description, time of onset and duration, intensity assessment, resolution, and action to be taken if applicable.

We expect mild adverse events such as fatigue and dizziness based on our feasibility study and the literature. These mild events usually are reported during the first week and regress spontaneously in a few days without interrupting the treatment. Safety outcomes include blood pressure monitoring every day from the screening period to the end of the final observation week. In addition, the patients will be monitored for heart rate, temperature, and weight at the beginning and the end of each period and the end of the observation week. Also, the blood formula will be checked at the beginning and the end of both study periods. Based on our precedent pilot study results and the safety profile of nabiximols, we don't expect adverse events affecting laboratory parameters.

### 2.7. Randomization, blinding, withdrawal

The participants will be randomized 1:1 between the sequence medical cannabinoids–placebo and the sequence placebo–medical cannabinoids (A→B and B→A). Patients, families, health professionals, and the study team will be blinded. The statistician (FC) generates the randomization list and provides it to Cannapharm AG, who will prepare study kits and complete the list with the batch numbers. The treatments are packed in sequentially numbered kits: each kit contains the placebo and the active drug in a clearly defined sequence, named periods 1 and 2, respecting the randomization list. The kits will be allocated in their numbered order to the patients. The study team will not have access to the list until the end of the study, except in an emergency. The blood sample analysis results concerning the blood concentration of THC, its derivatives, and CBD will be kept by the laboratory and communicated to the study team at the end of the second period to avoid demasking the patients following the observation of the presence of cannabinoid derivatives. Participants may withdraw from the study at any time and for any reason, and withdrawn patients won't be replaced.

Daily blood pressure measurement or sampling failure won't be considered a protocol violation. In fact, due to the particularity of our population, a refusal of the measurements and samplings may occur. Also, the failure to collect the family's impression of change won't be considered a protocol violation because some patients don't have relatives to provide such feedback.

### 2.8. Statistical consideration

Based on our feasibility study's results, the hypothesis is that medical cannabinoids will improve neuropsychiatric symptoms of patients with severe dementia living in a long-term care facility. The Cohen Mansfield agitation scale average score expected in patients before entering the study is 70 points. Therefore, the expected improvement with the treatment is a reduction of 20 points, with a between-patient standard deviation of 20 points and a within-patient standard deviation of 15 points. The number of patients to enroll, considering an alpha level of 5% and a power of 90%, is 14. Still, we planned to enroll 24 patients counting a 60% attrition rate. The high “attrition rate” in the power calculation is intended as a conservative measure because of the age and advanced illness of the trial participants and the potential risk of adverse events leading to trial dropouts. Moreover, the cross-over design is associated with more dropouts due to double duration, resulting in the loss of all information about the dropout participant due to the within-subjects analysis (60).

The changes from the baseline scores for primary and secondary endpoints will be evaluated by paired *t*-test, and the tolerability and safety data will be evaluated descriptively. Analysis with mixed models will be applied and defined in the statistical analysis plan finalized before the database lock. Subgroup analysis according to demographic criteria will be evaluated on an exploratory basis.

### 2.9. Data safety monitoring board (DSMB)

A Data Safety Monitoring Board will be established to safeguard the interests of trial participants by assessing the safety of the interventions during the trial and monitoring the study's overall conduct. The DSMB will be advisory to the Principal Investigator and the Sponsor, who are responsible for decisions regarding the trial. The DSMB will provide recommendations about modifying, continuing, or stopping the study and will function independently of all other individuals and bodies associated with the conduct of the trial. It will be composed of at least three voting members, who will be independent of the study and collectively have experience in the clinical area of interest, biostatistics, and randomized clinical trials. A quorum will require at least two members.

## 3. Discussion and conclusion

Dementia and its associated problems are increasing worldwide, and there is a lack of adequate and well-tolerated medications. A few clinical trials, with significant variations in settings and heterogeneity in study designs, suggest cannabinoids might be of interest (46). We conducted a prospective observational study to evaluate the feasibility and safety of using a standardized THC/CBD oral oil formulation to help treat BPSD in patients with severe dementia (55). The study results and the experience gained stand as the base for this randomized clinical trial whose study settings encounter overlapping difficulties (38, 61) starting from patient's consent. Patients with severe dementia cannot consent to study participation, so the consent must come from relatives and families. A parallel sociological evaluation showed that families were very supportive of participation and proud of contributing to advancement in research, feeling that their relatives had not been abandoned (62, 63). During the observational study, the patients weren't showing discomfort while receiving the medication. They instead seemed more comfortable, relaxed, in contact with family members and staff, and more willing to receive basic care. Also, the team was supportive of the experiment and is now eager to participate in the RCT.

The observational study gave us an indication of the clinical trial outcomes, and we designed primary and secondary endpoints based on previous results. Still, one relevant observation was concerning concomitant drug deprescription. Physicians were able to deprescribe pain and psychotropic medications during the observational trial in 84% of patients. Pain drugs could be reduced in 42% of patients. We believe pain assessment was missing data from the panel of evaluations in the observational study, and it could provide additional information on the overall clinical aspects. The Doloplus score is a validated observational assessment scale (13), and the study staff and the long-term facilities health professionals are used to it, considering that the knowledge of the scale is essential for the correct outcome measurement (64).

Several challenges have been identified in the proposed study, also considering Martin et al. suggestions for clinical trials with cannabis medicines (61). Primarily, we selected the same cannabis strain of the observational study and the same manufacturer who can also guarantee full quality control compliance. The quality certification and the stability of the natural oil are significant in assessing the efficacy of cannabinoids. The medical cannabis dosage titration and daily observation were attentively evaluated, especially considering patients’ reduced ability to communicate discomfort. Also, post-trial access was an essential aspect of the study setup, wanting to be able to provide medical cannabis treatment when asked by the physicians without additional costs. All these aspects represent strengths in the study design, together with the pharmacokinetic evaluation. The therapeutic drug monitoring and assessing cytochromes enzymatic activity is complex to organize in such a population, needing careful planning and coordination. Nevertheless, it is essential, especially considering the possibility of poly-medication, which is common to these patients, and the lack of reliable scientific data in the literature.

The principal limitation of this study is linked to the population. The cross-over design risks extending the length of the study, and patients might drop out between the two phases. Also, the recruitment might be prolonged by the several co-morbidities patients are experimenting with and the possibility of failing to respect inclusion and non-inclusion criteria. Those aspects have been anticipated in the sample size by calculating a significant attrition rate and choosing to recruit patients in five long-term facilities.

To our knowledge, this is one of the first studies to study the efficacy of cannabinoids in improving painful symptoms during severe dementia disorders in patients living in long-term care facilities. To date, one similar study is ongoing in Australia (65). Thus, the importance to confirm in different study settings and countries the efficacy of natural cannabinoids in treating behavioral symptoms and pain.

## Data Availability

The original contributions presented in the study are included in the article, further inquiries can be directed to the corresponding author.

## References

[1] Available at: https://www.who.int/news-room/fact-sheets/detail/dementia (Accessed October 2022).

[2] Leyhe T Jucker M Nef T Sollberger M Riese F Haba-Rubio J Conference report: dementia research and care and its impact in Switzerland. Swiss Med Wkly. (2020) 150:w20376. 10.4414/smw.2020.20376 33277912

[3] http://www.alz.ch/index.php/des-faits-et-des-chiffres.html.

[4] Alzheimer Schweiz. Demenz in der Schweiz: 2019, Zahlen und Fakten (2020). Available at: https://www.alzheimer-schweiz.ch/.

[5] McMinn B Draper B. Vocally disruptive behavior in dementia: development of an evidence based practice guideline. Aging Ment Health. (2005) 9:16–24. 10.1080/13607860512331334068 15841828

[6] Steinberg M Shao H Zandi P Lyketsos CG Welsh-Bohmer KA Norton MC Point and 5-year period prevalence of neuropsychiatric symptoms in dementia: the cache county study. Int J Geriatr Psychiatry. (2008) 23(2):170–7. 10.1002/gps.1858 17607801 PMC2932652

[7] McKeith I Cummings J. Behavioral changes and psychological symptoms in dementia disorders. Lancet Neurol. (2005) 4:735–42. 10.1016/S1474-4422(05)70219-2 16239180

[8] Kales HC Gitlin LN Lyketsos CG. Assessment and management of behavioral and psychological symptoms of dementia. Br Med J. (2015) 350:h369. 10.1136/bmj.h369 25731881 PMC4707529

[9] Kunik ME Snow AL Davila JA Steele AB Balasubramanyam V Doody RS Causes of aggressive behavior in patients with dementia. J Clin Psychiatry. (2010) 71(9):1145–52. 10.4088/JCP.08m04703oli 20361896

[10] van Kooten J Binnekade TT van der Wouden JC Stek ML Scherder EJ Husebø BS A review of pain prevalence in Alzheimer’s, vascular, frontotemporal and lewy body dementias. Dement Geriatr Cogn Disord. (2016) 41(3-4):220–32. 10.1159/000444791 27160163

[11] Ahn H Horgas A. The relationship between pain and disruptive behaviors in nursing home residents with dementia. BMC Geriatr. (2013) 13:14. 10.1186/1471-2318-13-14 23399452 PMC3573898

[12] Boltz M Resnick B Kuzmik A Mogle J Jones JR Arendacs R Pain incidence, treatment, and associated symptoms in hospitalized persons with dementia. Pain Manag Nurs. (2021) 22(2):158–63. 10.1016/j.pmn.2020.08.002 32921569 PMC7943650

[13] Lefebvre-Chapiro S. The Doloplus-2 scale–evaluating pain in the elderly. Eur J Palliat Care. (2001) 8:191–4. http://www.haywardpublishing.co.uk/journal_search_results.aspx?JournalID=4&sw=0&yrFrom=2000&yrTo=2001&sa=Lefebvre&ef=False&notw=0&alw=True

[14] Pautex S Herrmann FR Michon A Giannakopoulos P Gold G. Psychometric properties of the Doloplus-2 observational pain assessment scale and comparison to self-assessment in hospitalized elderly. Clin J Pain. (2007) 23(9):774–9. 10.1097/AJP.0b013e318154b6e3 18075404

[15] Gareri P Segura-García C Manfredi VG Bruni A Ciambrone P Cerminara G Use of atypical antipsychotics in the elderly: a clinical review. Clin Interv Aging. (2014) 9:1363–73. 10.2147/CIA.S63942. PMID: ; PMCID: . 25170260 PMC4144926

[16] Schneider LS Pollock VE Lyness SA. A meta-analysis of controlled trials of neuroleptic treatment in dementia. J Am Geriatr Soc. (1990) 38:553–63. 10.1111/j.1532-5415.1990.tb02407.x 1970586

[17] Barrett-Connor E Edelstein SL Corey-Bloom J Wiederholt WC. Weight loss precedes dementia in community-dwelling older adults. J Am Geriatr Soc. (1996) 44:1147–52. 10.1111/j.1532-5415.1996.tb01362.x 8855991

[18] MacCallum CA Russo EB. Practical considerations in medical cannabis administration and dosing. EJIM. (2018) 49:12–9. 10.1016/j.ejim.2018.01.004 29307505

[19] Devinsky O Cilio MR Cross H Fernandez-Ruiz J French J Hill C Cannabidiol: pharmacology and potential therapeutic role in epilepsy and other neuropsychiatric disorders. Epilepsia. (2014) 55(6):791–802. 10.1111/epi.12631 24854329 PMC4707667

[20] LaVigne JE Hecksel R Keresztes A Streicher JM. Cannabis sativa terpenes are cannabimimetic and selectively enhance cannabinoid activity. Sci Rep. (2021) 11(1):8232. 10.1038/s41598-021-87740-8 33859287 PMC8050080

[21] Koehler J. Who benefits most from THC:CBD spray? Learning from clinical experience. Eur Neurol. (2014) 71(Suppl 1):10–5. 10.1159/000357743 24457847

[22] Meyer T Funke A Münch C Kettemann D Maier A Walter B Real world experience of patients with amyotrophic lateral sclerosis (ALS) in the treatment of spasticity using tetrahydrocannabinol:cannabidiol (THC:CBD). BMC Neurol. (2019) 19(1):222. 10.1186/s12883-019-1443-y 31493784 PMC6732193

[23] Beal JE Olson R Laubenstein L Morales JO Bellman P Yangco B Dronabinol as a treatment for anorexia associated with weight loss in patients with AIDS. J Pain Symptom Manage. (1995) 10(2):89–97. 10.1016/0885-3924(94)00117-4 7730690

[24] Mathew RJ Wilson WH Chiu NY Turkington TG Degrado TR Coleman RE. Regional cerebral blood flow and depersonalization after tetrahydrocannabinol administration. Acta Psychiatr Scand. (1999) 100(1):67–75. 10.1111/j.1600-0447.1999.tb10916.x 10442442

[25] Bergamaschi MM Queiroz RH Chagas MH de Oliveira DC De Martinis BS Kapczinski F Cannabidiol reduces the anxiety induced by simulated public speaking in treatment-naïve social phobia patients. Neuropsychopharmacology. (2011) 36(6):1219–26. 10.1038/npp.2011.6 21307846 PMC3079847

[26] Bisogno T Hanus L De Petrocellis L Tchilibon S Ponde DE Brandi I Molecular targets for cannabidiol and its synthetic analogues: effect on vanilloid VR1 receptors and on the cellular uptake and enzymatic hydrolysis of anandamide. Br J Pharmacol. (2001) 134(4):845–52. 10.1038/sj.bjp.0704327 11606325 PMC1573017

[27] Magen I Avraham Y Ackerman Z Vorobiev L Mechoulam R Berry EM. Cannabidiol ameliorates cognitive and motor impairments in bile-duct ligated mice via 5-HT1A receptor activation. Br J Pharmacol. (2010) 159(4):950–7. 10.1111/j.1476-5381.2009.00589.x 20128798 PMC2829220

[28] Watanabe K Kayano Y Matsunaga T Yamamoto I Yoshimura H. Inhibition of anandamide amidase activity in mouse brain microsomes by cannabinoids. Biol Pharm Bull. (1996) 19(8):1109–11. 10.1248/bpb.19.1109 8874830

[29] Rakhshan F Day TA Blakely RD Barker EL. Carrier-mediated uptake of the endogenous cannabinoid anandamide in RBL-2H3 cells. J Pharmacol Exp Ther. (2000) 292(3):960–7. https://jpet.aspetjournals.org/content/292/3/960 10688610

[30] Chung H Fierro A Pessoa-Mahana CD. Cannabidiol binding and negative allosteric modulation at the cannabinoid type 1 receptor in the presence of delta-9-tetrahydrocannabinol: an in silico study. PLoS One. (2019) 14(7):e0220025. 10.1371/journal.pone.0220025 31335889 PMC6650144

[31] Niesink RJ van Laar MW. Does cannabidiol protect against adverse psychological effects of THC? Front Psychiatry. (2013) 4(130). 10.3389/fpsyt.2013.00130 24137134 PMC3797438

[32] Karniol IG Shirakawa I Kasinski N Pfeferman A Carlini EA. Cannabidiol interferes with the effects of delta 9 - tetrahydrocannabinol in man. Eur J Pharmacol. (1974) 28(1):172–7. 10.1016/0014-2999(74)90129-0 4609777

[33] Fadda P Robinson L Fratta W Pertwee RG Riedel G. Differential effects of THC- or CBD-rich cannabis extracts on working memory in rats. Neuropharmacology. (2004) 47(8):1170–9. 10.1016/j.neuropharm.2004.08.009 15567426

[34] Russo E Guy GW. A tale of two cannabinoids: the therapeutic rationale for combining tetrahydrocannabinol and cannabidiol. Med Hypotheses. (2006) 66(2):234–46. 10.1016/j.mehy.2005.08.026 16209908

[35] Nicholson AN Turner C Stone BM Robson PJ. Effect of Δ-9-tetrahydrocannabinol and cannabidiol on nocturnal sleep and early-morning behavior in young adults. J Clin Psychopharmacol. (2004) 24(3):305–13. 10.1097/01.jcp.0000125688.05091.8f 15118485

[36] Englund A Oliver D Chesney E Chester L Wilson J Sovi S Does cannabidiol make cannabis safer? A randomised, double-blind, cross-over trial of cannabis with four different CBD:THC ratios. Neuropsychopharmacology. (2022) 48:869–76. 10.1038/s41386-022-01478-z. PMID: 36380220 PMC10156730

[37] Ben-Shabat S Fride E Sheskin T Tamiri T Rhee MH Vogel Z An entourage effect: inactive endogenous fatty acid glycerol esters enhance 2-arachidonoyl-glycerol cannabinoid activity. Eur J Pharmacol. (1998) 353(1):23–31. 10.1016/s0014-2999(98)00392-6 9721036

[38] Russo EB. Current therapeutic cannabis controversies and clinical trial design issues. Front Pharmacol. (2016) 7(309). 10.3389/fphar.2016.00309 PMC5022003 27683558

[39] Russo EB. The case for the entourage effect and conventional breeding of clinical cannabis: no “strain,” no gain. Front Plant Sci. (2019) 9:1969. 10.3389/fpls.2018.01969 30687364 PMC6334252

[40] Johnson JR Burnell-Nugent M Lossignol D Ganae-Motan ED Potts R Fallon MT. Multicenter, double-blind, randomized, placebo-controlled, parallel-group study of the efficacy, safety, and tolerability of THC:CBD extract and THC extract in patients with intractable cancer-related pain. J Pain Symptom Manage. (2010) 39(2):167–79. 10.1016/j.jpainsymman.2009.06.008 19896326

[41] Berman JS Symonds C Birch R. Efficacy of two cannabis based medicinal extracts for relief of central neuropathic pain from brachial plexus avulsion: results of a randomised controlled trial. Pain. (2004) 112(3):299–306. 10.1016/j.pain.2004.09.013 15561385

[42] Fisher E Moore RA Fogarty AE Finn DP Finnerup NB Gilron I Cannabinoids, cannabis, and cannabis-based medicine for pain management: a systematic review of randomised controlled trials. Pain. (2021) 162(Suppl 1):S45–S66. 10.1097/j.pain.0000000000001929 32804836

[43] Kilcher G Zwahlen M Ritter C Fenner L Egger M. Medical use of cannabis in Switzerland: analysis of approved exceptional licences. Swiss Med Wkly. (2017) 147:w14463. 10.4414/smw.2017.14463 28695562

[44] Aso E Ferrer I. Cannabinoids for treatment of Alzheimer’s disease: moving toward the clinic. Front Pharmacol. (2014) 5:37. 10.3389/fphar.2014.00037 24634659 PMC3942876

[45] Liu CS Chau SA Ruthirakuhan M Lanctôt KL Herrmann N. Cannabinoids for the treatment of agitation and aggression in Alzheimer’s disease. CNS Drugs. (2015) 29(8):615–23. 10.1007/s40263-015-0270-y 26271310

[46] Hillen JB Soulsby N Alderman C Caughey GE. Safety and effectiveness of cannabinoids for the treatment of neuropsychiatric symptoms in dementia: a systematic review. Ther Adv Drug Saf. (2019) 10:2042098619846993. 10.1177/2042098619846993 31205674 PMC6535742

[47] Lucas CJ Galettis P Schneider J. The pharmacokinetics and the pharmacodynamics of cannabinoids. Br J Clin Pharmacol. (2018) 84(11):2477–82. 10.1111/bcp.13710 30001569 PMC6177698

[48] Stout SM Cimino NM. Exogenous cannabinoids as substrates, inhibitors, and inducers of human drug metabolizing enzymes: a systematic review. Drug Metab Rev. (2014) 46(1):86–95. 10.3109/03602532.2013.849268 24160757

[49] Bansal S Maharao N Paine MF Unadkat JD. Predicting the potential for cannabinoids to precipitate pharmacokinetic drug interactions via reversible inhibition or inactivation of major cytochromes P450. Drug Metab Dispos. (2020) 48(10):1008–17. 10.1124/dmd.120.000073 32587099 PMC7543485

[50] Jiang R Yamaori S Okamoto Y Yamamoto I Watanabe K. Cannabidiol is a potent inhibitor of the catalytic activity of cytochrome P450 2C19. Drug Metab Pharmacokinet. (2013) 28(4):332–8. 10.2133/dmpk.dmpk-12-rg-129 23318708

[51] Yamaori S Koeda K Kushihara M Hada Y Yamamoto I Watanabe K. Comparison in the in vitro inhibitory effects of major phytocannabinoids and polycyclic aromatic hydrocarbons contained in marijuana smoke on cytochrome P450 2C9 activity. Drug Metab Pharmacokinet. (2012) 27(3):294–300. 10.2133/dmpk.dmpk-11-rg-107 22166891

[52] Nasrin S Watson CJW Perez-Paramo YX Lazarus P. Cannabinoid metabolites as inhibitors of major hepatic CYP450 enzymes, with implications for cannabis-drug interactions. Drug Metab Dispos. (2021) 49(12):1070–80. 10.1124/dmd.121.000442 34493602 PMC11022895

[53] Engels FK de Jong FA Sparreboom A Mathot RA Loos WJ Kitzen JJ Medicinal cannabis does not influence the clinical pharmacokinetics of irinotecan and docetaxel. Oncologist. (2007) 12(3):291–300. 10.1634/theoncologist.12-3-291 17405893

[54] Gaston TE Bebin EM Cutter GR Liu Y Szaflarski JP, UAB CBD program. Interactions between cannabidiol and commonly used antiepileptic drugs. Epilepsia. (2017) 58(9):1586–92. 10.1111/epi.13852 28782097

[55] Pautex S Bianchi F Daali Y Augsburger M de Saussure C Wampfler J Cannabinoids for behavioral symptoms in severe dementia: safety and feasibility in a long-term pilot observational study in nineteen patients. Front Aging Neurosci. (2022) 14(957665). 10.3389/fnagi.2022.957665 36247984 PMC9557769

[56] Cohen-Mansfield J Deutsch LH. Agitation: subtypes and their mechanisms. Semin Clin Neuropsychiatry. (1996) 1:325–39. 10.1053/SCNP00100325 10320435

[57] Cummings JL Mega M Gray K Rosenberg-Thompson S Carusi DA Gornbein J. The neuropsychiatric inventory: comprehensive assessment of psychopathology in dementia. Neurology. (1994) 44:2308–14. 10.1212/WNL.44.12.2308 7991117

[58] Richards M Marder K Cote L Mayeux R. Interrater reliability of the unified Parkinson’s disease rating scale motor examination. Movements Disorders. (1994) 9(1):89–91. 10.1002/mds.870090114 8139610

[59] Bosilkovska M Samer CF Déglon J Rebsamen M Staub C Dayer P Geneva cocktail for cytochrome P450 and P-glycoprotein activity assessment using dried blood spots. Clin Pharmacol Ther. (2014) 96(3):349–59. 10.1038/clpt.2014.83 24722393 PMC4151019

[60] Senn S. Cross-over trials in clinical research. Chichester: Wiley (1993). ISBN 0-471-93493-3.

[61] Martin JH Hill C Walsh A Efron D Taylor K Kennedy M Clinical trials with cannabis medicines—guidance for ethics committees, governance officers and researchers to streamline ethics applications and ensuring patient safety: considerations from the Australian experience. Trials. (2020) 21(932). 10.1186/s13063-020-04862-6 PMC7673085 33203469

[62] Revol A. Prescription de cannabis à usage thérapeutique pour les personnes âgées atteintes de démence: l’engouement des proches aidants. Psychotropes. (2019) 25:129–49. 10.3917/psyt.252.0129

[63] Revol A. Au moins on tente quelque chose “: cannabis thérapeutique et lien social. Gérontologie et société. (2022) 44(167):215–30. 10.3917/gs1.167.0215

[64] Rostad HM Utne I Grov EK Puts M Halvorsrud L. Measurement properties, feasibility and clinical utility of the Doloplus-2 pain scale in older adults with cognitive impairment: a systematic review. BMC Geriatr. (2017) 17(1):257. 10.1186/s12877-017-0643-9 29096611 PMC5667437

[65] Timler A Bulsara C Bulsara M Vickery A Smith J Codde J. Use of cannabinoid-based medicine among older residential care recipients diagnosed with dementia: study protocol for a double-blind randomised crossover trial. Trials. (2020) 21(1):188. 10.1186/s13063-020-4085-x 32059690 PMC7023743

